# Role of Right Ventricular Global Longitudinal Strain in Predicting Early and Long-Term Mortality in Cardiac Resynchronization Therapy Patients

**DOI:** 10.1371/journal.pone.0143907

**Published:** 2015-12-23

**Authors:** Vivien Klaudia Nagy, Gábor Széplaki, Astrid Apor, Valentina Kutyifa, Attila Kovács, Annamária Kosztin, Dávid Becker, András Mihály Boros, László Gellér, Béla Merkely

**Affiliations:** Heart and Vascular Center, Semmelweis University, Budapest, Hungary; Fondazione G. Monasterio, ITALY

## Abstract

**Background:**

Right ventricular (RV) dysfunction has been associated with poor prognosis in chronic heart failure (HF). However, less data is available about the role of RV dysfunction in patients with cardiac resynchronization therapy (CRT). We aimed to investigate if RV dysfunction would predict outcome in CRT.

**Design:**

We enrolled prospectively ninety-three consecutive HF patients in this single center observational study. All patients underwent clinical evaluation and echocardiography before CRT and 6 months after implantation. We assessed RV geometry and function by using speckle tracking imaging and calculated strain parameters. We performed multivariable Cox regression models to test mortality at 6 months and at 24 months.

**Results:**

RV dysfunction, characterized by decreased RVGLS (RV global longitudinal strain) [10.2 (7.0–12.8) vs. 19.5 (15.0–23.9) %, p<0.0001] and RVFWS (RV free wall strain) [15.6 (10.0–19.3) vs. 17.4 (10.5–22.2) %, p = 0.04], improved 6 months after CRT implantation. Increasing baseline RVGLS and RVFWS predicted survival independent of other parameters at 6 months [hazard ratio (HR) = 0.37 (0.15–0.90), p = 0.02 and HR = 0.42 (0.19–0.89), p = 0.02; per 1 standard deviation increase, respectively]. RVGLS proved to be a significant independent predictor of mortality at 24 months [HR = 0.53 (0.32–0.86), p = 0.01], and RVFWS showed a strong tendency [HR = 0.64 (0.40–1.00), p = 0.05]. The 24-month survival was significantly impaired in patients with RVGLS below 10.04% before CRT implantation [area under the curve = 0.72 (0.60–0.84), p = 0.002, log-rank p = 0.0008; HR = 5.23 (1.76–15.48), p = 0.003].

**Conclusions:**

Our findings indicate that baseline RV dysfunction is associated with poor short-term and long-term prognosis after CRT implantation.

## Introduction

The prevalence of chronic heart failure (HF) increases and despite modern therapy, mortality is high, which causes enormous economic burden to the society [1]. Cardiac resynchronisation therapy (CRT) is effective in patients with drug refractory, advanced heart failure by reducing mortality and improving left ventricular systolic function, and quality of life [2, 3]^.^ Still, 20–30% of patients do not respond to CRT as adequately as would be expected [4]. Therefore, identifying patients who would not benefit from CRT is a burning issue.

Previous studies have shown that right ventricular (RV) dysfunction, besides left ventricular impairment, is an important prognostic factor in patients with moderate to severe chronic heart failure [5, 6]. However, the role of RV function in CRT patients has not been fully evaluated [7, 8].

Even though echocardiography is the fundamental method for diagnosis establishment and prognosis evaluation in HF, RV function is infrequently evaluated, due to the relative complexity of right heart morphology and function. A possible solution would be speckle tracking analysis, which quantifies both global and regional wall motion and deformation in a way that is relatively unaffected by cardiac translational motion and tethering [9].

To this aim, we investigated if baseline RV function assessed by speckle tracking imaging would predict the short- and long-term mortality of chronic heart failure patients undergoing CRT.

## Methods

### Study population

A cohort of 131 consecutive, chronic HF patients with CRT indication were enrolled between September 2009 and December 2010 to our prospective, single-centre observational study. In accordance with the current and recent guidelines, the inclusion criteria included: symptomatic heart failure [New York Heart Association (NYHA) II-IVa functional class] treated with maximal tolerated medical therapy for at least 3 months prior to inclusion; LV ejection fraction (LVEF) below 35%; QRS duration wider than 120 ms (measured on surface electrocardiogram using the widest QRS complex from II, V1 and V6 leads) [10–13]. Exclusion criteria were malignancies, diagnosed inflammatory diseases and primary genetic cardiomyopathies. All patients provided their written informed consent. The study adhered to the Declaration of Helsinki and was approved by the local ethics committee of the Semmelweis University.

The present echocardiographic sub-study included 93 patients, who consented to undergo detailed echocardiographic examination (S1 Dataset).

### Study protocol and outcomes

Prior to CRT implantation, at the baseline visit we examined the physical status of the patients, recorded medical history and performed ECG analysis and detailed echocardiography. At 6 months follow-up we carried out echocardiography and clinical status assessment. Twenty-four months after CRT implantation we evaluated clinical status. The primary end-point of the study was all-cause mortality at the 6 as well as the 24 months follow-up.

### Device implantation

All patients underwent successful CRT-P (pacemaker only CRT) or CRT-D (CRT with implantable cardioverter defibrillator) implantation. The right atrial and right ventricular leads were positioned in the right appendage and mid interventricular septum or in the RV apex. Left ventricular lead positioning was performed under guidance of venography, and when accessible lateral or postero-lateral cardiac veins were chosen. Implanted devices included Contak Renewal (Guidant Corp., Arden Hills, MN, USA), Cognis (Boston Scientific USA), Stratos, Lumax (Biotronik, Berlin, Germany) and Insync III (Medtronic Inc., Minneapolis, MN, USA). We implanted CRT-Ds to 46% of the patients. CRT-D was indicated in case of those patients who suffered previously documented ventricular arrhythmias and whenever it was indicated by the referring physician [14].

### Echocardiography and Strain Imaging

We performed transthoracic echocardiography with a commercially available cardiovascular ultrasound system (iE33, Philips Healthcare, Best, The Netherlands) and used S5-1 transducer (Philips Healthcare, Best, The Netherlands) for the 2D and Doppler imaging. Standard parasternal short-, long-axis and apical views were acquired in left lateral supine position. We repeated all measurements three times in separate cardiac cycles and calculated the average value of the results for further analysis.

We assessed left ventricular end-diastolic volume (LVEDV), left ventricular end-systolic volume (LVESV), LVEF and left atrial volume (LAV) using the modified Simpson’s biplane method. Wall motion abnormalities were detected in ischaemic patients using a model that divides the left ventricle into 5 segments (septal, lateral, anterior, inferior, and apical) and akinetic wall segment abnormalities were considered for further analysis.

Guidelines proposed by the American College of Echocardiography were used to evaluate right ventricular dimensions and function [15]. Right atrial (RA) area measurement was performed using planimetry, and RA volume was calculated using the modified Simpson’s method. We traced the endocardial borders of RV end-diastolic and end-systolic areas (RVEDA, RVESA) manually and defined right ventricular fractional area change (RVFAC) by using the [(RVEDA—RVESA)/RVEDA x 100] formula. The base-to-apex shortening of the RV during systole was represented by tricuspid annular plane systolic excursion (TAPSE), which was recorded on M-mode, guided by two-dimensional (2D) echocardiography.

We determined semiquantitatively both mitral (MI) and tricuspid insufficiency (TI) on a 4 point scale based on the maximal distance of regurgitant jet originating from the valve orifice. Pulmonary artery systolic pressure (PASP) was evaluated using continuous wave (CW) Doppler measurement of maximal transtricuspid regurgitant flow velocity. Left ventricular diastolic function was characterised by the mitral inflow pattern using pulsed-wave (PW) Doppler: peak velocities of E wave [with deceleration time (DT)] and A wave were measured, then the velocity ratio (E/A) was calculated. We performed tissue Doppler imaging (TDI) to measure the early (E’) diastolic annular velocity at the lateral corner of the mitral annulus. Mitral E velocity, corrected for the influence of relaxation (i.e., the E/E’ ratio), was calculated to estimate left ventricular filling pressure.

For RV 2D speckle tracking analysis, ECG-gated digital cine-loops were obtained from apical four-chamber view with a frame rate between 60 and 100 frames/sec. We achieved a better visualisation of the right ventricle with an off-axis apical four-chamber view. Special care was taken to obtain the best visualisation of the whole RV for a more precise delineation of the endocardial borders.

Off-line analysis of the acquired cine-loops was performed by an independent cardiologist expert using a commercially available software (QLab, version 9.0, Philips, Andover, MA, USA). To assess the regional and global RV systolic function, a 7-segment RV model was deployed (basal RV free wall, mid RV free wall, apical RV free wall, apex, apical septum, mid septum and basal septum). We calculated RV global longitudinal strain (GLS) as the average value of the peak systolic strain of the segments. RV free wall strain was generated by making the average value of the peak systolic strains of the 3 free RV wall segments. We would like to highlight that the strain parameters are presented as the absolute values of the measured ones for clarity and better understanding. We excluded those RV wall segments that were impossible to track adequately and in these cases we calculated strains as the average of the acquired segments [16].

### Statistical analysis

A two-tailed p-value <0.05 was considered statistically significant and data analysis was carried out by using IBM SPSS 21 (Apache Software Foundation, USA) and Graphpad Prism 6.03 (GraphPad Softwares Inc, USA).

The variables reported in this study deviated from normal distribution (analysed by Shapiro-Wilk test). Categorical variables are presented as event numbers and percentage, while continuous variables are expressed as median with interquartile range. For group comparison we used the Mann-Whitney or Wilcoxon-test for continuous variables and the Chi-squared test for dichotomous variables. We performed receiver operating characteristics curve analysis to define optimal cut-off values.

We set up multivariable Cox regression models in a forward stepwise way: the first model (M1) included those variables, which were statistically significant in the univariate analysis; the second model (M2) comprised covariates with proven relevance in chronic heart failure. Continuous variables were ranked and standardized by one standard deviation increase (Z transformation). The proportional hazard assumption for continuous covariates was tested using Schoenfeld residuals [17].

Intra- and interobserver variability test for RV speckle tracking analysis was performed in 10 randomly selected patients using Lin correlation. All values exceeded 0.90 (intraobserver variability: RVGLS: 0.94, RVFWS: 0.92; interobserver variability: RVGLS: 0.91, RVFWS 0.90), suggesting good reliability and replicability.

## Results

### Baseline clinical characteristics

The baseline clinical characteristics of the 93 patients are shown in Table 1. The median age was 67 (60–72) years, the majority were male (78%) and half of the patients (49%) presented with heart failure of ischemic origin. Ninety percent of the patients showed left bundle branch block (LBBB) in ECG.

**Table 1:** Patient baseline clinical characteristics and univariate Cox regression analysis for prediction of mortality at 6 and 24 months after CRT-implantation.

		mortality at 6 months				mortality at 24 months			
	Total cohort	HR	95% CI	χ2	p	HR	95% CI	χ^2^	p
**Age (years)**	67 (60–72)	1.25	0.58–2.66	0.34	0.55	1.07	0.69–1.67	0.10	0.74
**Male**	72 (78%)	2.23	0.44–11.75	0.96	0.33	1.62	0.45–5.83	0.55	0.46
**Ischaemic ethiology**	46 (49%)	1.29	0.34–4.82	0.15	0.69	1.97	0.82–4.71	2.35	0.12
**NYHA III/IV**	81 (87%)	1.19	0.15–9.57	0.02	0.86	3.22	0.43–23.99	1.30	0.25
**LBBB**	84 (90%)	0.45	0.09–2.18	0.96	0.32	0.42	0.15–1.16	2.78	0.09
**QRS-duration (ms)**	160 (150–180)	0.41	0.19–0.91	4.77	**0.02**	0.59	0.36–0.97	4.30	**0.03**
**CRT-D**	43 (46%)	1.47	0.39–5.48	0.33	0.56	1.27	0.55–2.95	0.32	0.56
**Optimal lead position**	68 (73%)	1.21	0.25–5.82	0.05	0.81	1.37	0.50–3.75	0.37	0.54
**Diabetes mellitus**	29 (31%)	1.09	0.27–4.39	0.01	0.89	1.92	0.83–4.64	2.35	0.12
**Hypertension**	48 (51%)	0.45	0.11–1.81	1.24	0.26	0.62	0.26–1.46	1.16	0.28
**Atrial fibrillation**	26 (27%)	1.25	0.31–5.00	0.10	0.75	1.28	0.51–3.18	0.29	0.58
**BB**	85 (91%)	0.15	0.03–0.62	6.89	**0.009**	0.49	0.14–1.67	1.27	0.25
**ACEi/ARB**	88 (94%)	0.24	0.05–1.19	3.01	0.08	0.56	0.13–2.44	0.58	0.44
**Diuretics**	76 (81%)	1.79	0.22–14.31	0.30	0.58	2.15	0.50–9.28	1.07	0.30
**AA**	69 (74%)	0.25	0.06–0.93	4.21	**0.04**	1.03	0.38–2.81	0.00	0.94

Hazard ratios refer to 1 standard deviation increase in case of continuous variables and presence versus absence in case of categorical variables. HR: hazard ratio, CI: confidence interval, χ^2^: wald chi square, NYHA: New York Heart Association Classification, LBBB: left bundle branch block, CRT-D: cardiac resynchronisation therapy with defibrillator, BB: beta-blocker, ACEi/ARB: angiotensin convertase inhibitor/angiotensin receptor blocker, AA: aldosterone antagonist, LVEF: left ventricular ejection fraction, LVEDV: left ventricular end-diastolic volume, LVESV: left ventricular end-systolic volume, MI: mitral insufficiency.

In our population, shorter QRS duration predicted death 6 months (n = 9) and 24 months (n = 22) after CRT implantation and the lack of beta-blocker (BB) and aldosterone antagonist (AA) intake was associated with poor 6 months prognosis (Table 1).

### Left-side echocardiographic parameters

We observed reduced LVEF, marked left ventricular and left atrial dilatation in our patients with moderate mitral regurgitation, as shown in Table 2. As the result of cardiac resynchronization, a significant improvement was seen in LVEF [29.7 (24.2–32.7) vs. 39.4 (32.3–44.7) %, p<0.0001] 6 months after implantation that was characterized by remarkable reduction in the LVEDV [238.7 (185.2–293.9) vs. 211.5 (159.4–255.4) ml, p<0.0001) and LVESV [173.8 (135.5–217.1) vs. 129.9 (90.1–174.5) ml, p<0.0001]. CRT also decreased LAV [95.5 (71.2–121.9) vs. 83.1 (61.7–107.4) ml, p = 0.02], which was a significant predictor of mortality at 24 months (Table 2).

**Table 2:** Left ventricular dimensions and function before CRT implantation and univariate Cox regression analysis in case of mortality at 6 and 24 months.

		mortality at 6 months				mortality at 24 months			
	Total cohort	HR	95% CI	χ2	p	HR	95% CI	χ^2^	p
**LVEF (%)**	29.7 (24.2–32.7)	1.08	0.54–2.13	0.05	0.82	0.83	0.55–1.26	0.71	0.39
**LVEDV (ml)**	238.7 (185.2–293.9)	0.67	0.31–1.42	1.07	0.30	0.69	0.43–1.11	2.31	0.12
**LVESV (ml)**	173.8 (135.5–217.1)	0.73	0.34–1.55	0.66	0.41	0.77	0.48–1.23	1.14	0.28
**LAV (ml)**	95.5 (71.2–121.9)	1.41	0.80–2.48	1.42	0.23	1.46	1.01–2.09	4.22	**0.04**
**MI**	2.0 (1.0–2.5)	1.19	0.57–2.49	0.22	0.63	1.48	0.95–2.31	3.03	0.08
**E/A**	1.08 (0.73–1.76)	1.36	0.66–2.79	0.73	0.39	1.22	0.81–1.83	0.93	0.33
**DT**	160.0 (129.7–200.0)	0.96	0.50–1.85	0.01	0.91	0.77	0.48–1.23	1.15	0.28
**E’**	8.3 (6.2–10.2)	1.08	0.54–2.14	0.04	0.82	0.90	0.57–1.40	0.21	0.64
**E/E’**	10.6 (8.0–14.0)	0.79	0.35–1.80	0.29	0.58	1.03	0.67–1.60	0.02	0.86

Hazard ratios refer to 1 standard deviation increase in case of continuous variables and presence versus absence in case of categorical variables. HR: hazard ratio, CI: confidence interval, χ^2^: wald chi square, LVEF: left ventricular ejection fraction, LVEDV: left ventricular end-diastolic volume, LVESV: left ventricular end-systolic function, LAV: left atrial volume, MI: mitral insufficiency, E: transmitral E wave, DT: E-wave deceleration time, E’: mitral lateral annulus tissue Doppler imaging E’ wave.

### Right-side echocardiographic parameters

The reduced RV function, characterized by decreased RVGLS [10.2 (7.0–12.8) vs. 19.5 (15.0–23.9) %, p<0.0001] and RVFWS [15.6 (10.0–19.3) vs. 17.4 (10.5–22.2) %, p = 0.04], improved 6 months after CRT implantation. However, conventional RV functional parameters, RVFAC [41.1 (30.85–51.8) vs. 42.7 (35.9–51.2) %, p = 0.74], and TAPSE [17.5 (14.0–22.0) vs. 18.2 (14.7–22.0) mm, p = 0.66] did not show statistically significant differences during the 6 months follow-up.

The univariate Cox regression analysis revealed an overall better prognosis in patients with increased baseline RVGLS and RVFWS, as shown in Table 3. Furthermore, patients showing elevated baseline RVFAC and TAPSE survived the 24 months post-implant period much better.

**Table 3:** Baseline RV morphological and functional parameters predictive of 6 months and 24 months mortality during univariate Cox regression analysis.

		mortality at 6 months				mortality at 24 months			
	Total cohort	HR	95% CI	χ2	p	HR	95% CI	χ2	p
**RVTD (mm)**	36.0 (32.0–43.0)	1.35	0.73–2.46	0.94	0.33	1.34	0.90–1.99	2.11	0.14
**RA area (cm** ^**2**^ **)**	21.3 (17.4–28.3)	1.03	0.54–1.97	0.01	0.91	1.38	0.95–2.01	2.91	0.08
**RAV (ml)**	68.6 (48.8–101.7)	0.96	0.49–1.88	0.01	0.91	1.34	0.94–1.91	2.72	0.09
**RVEDA (cm2)**	22.5 (18.2–27.8)	1.22	0.67–2.21	0.42	0.51	1.20	0.80–1.79	0.84	0.35
**RVESA (cm2)**	13.1 (9.1–17.3)	1.39	0.80–2.41	1.37	0.24	1.42	0.99–2.04	3.76	0.05
**RVFAC (%)**	41.1 (30.85–51.8)	0.55	0.28–1.08	2.97	0.08	0.54	0.35–0.83	7.72	**0.005**
**TAPSE (mm)**	17.5 (14.0–22.0)	0.60	0.27–1.36	1.46	0.22	0.53	0.33–0.86	6.53	**0.01**
**TI**	1.0 (0.5–2.0)	1.15	0.56–2.36	0.16	0.68	1.45	0.98–2.15	3.46	0.06
**PASP (Hgmm)**	27.2 (20.9–37.0)	1.25	0.67–2.33	0.51	0.47	1.27	0.87–1.85	1.58	0.20
**RVGLS (%)**	10.2 (7.0–12.8)	0.46	0.22–0.95	4.41	**0.036**	0.50	0.32–0.79	8.85	**0.003**
**RVFWS (%)**	15.6 (10.0–19.3)	0.470	0.24–0.93	4.70	**0.030**	0.58	0.38–0.88	6.56	**0.01**

Hazard ratios refer to 1 standard deviation increase. HR: hazard ratio, CI: confidence interval, χ^2^: wald chi square, OR: odds ratio, RVTD: right ventricular transversal diameter, RAV: right atrial volume, RVEDA: right ventricular end-diastolic area, RVESA: right ventricular end-systolic area, RVFAC: right ventricular fractional area change, TAPSE: tricuspid annular plane systolic excursion, TI: tricuspid insufficiency, PASP: pulmonary artery systolic pressure, RVGLS: right ventricular global longitudinal strain, RVFWS: right ventricular free wall strain.

### Multivariable analyses

In order to assess the independent effect of RV strain parameters on mortality, we set up a multivariable model (M1) adjusted to the significant clinical and left-side echocardiographic parameters from the univariate analysis (6 months: QRS, BB and AA intake; 24 months: QRS, LAV), as shown in Table 4.

**Table 4:** Multivariable Cox regression analysis of right ventricular functional parameters predicting mortality at 6 and 24 months. Model 1 (M1): those baseline clinical parameters were put in as co-variates, which were predictive of mortality during univariate analysis.

	M1								
	mortality at 6 months					mortality at 24 months			
	HR	95% CI	χ^2^	p		HR	95% CI	χ^2^	p
**RVGLS**	0.37	0.15–0.90	4.74	**0.02**	**RVGLS**	0.53	0.32–0.86	6.58	**0.01**
**RVFWS**	0.42	0.19–0.89	5.05	**0.02**	**RVFWS**	0.64	0.40–1.00	3.70	0.054
** **				** **	**RVFAC**	0.67	0.42–1.06	2.84	0.09
** **				** **	**TAPSE**	0.58	0.35–0.96	4.42	**0.03**

Hazard ratios refer to 1 standard deviation increase. Models were adjusted for QRS duration, intake of beta blocker and aldosterone antagonist in case of mortality at 6 months and QRS duration and left atrial volume in case of mortality at 24 months. HR: hazard ratio, CI: confidence interval, χ^2^: wald chi square, OR: odds ratio, RVGLS: right ventricular global longitudinal strain.

Increasing baseline RVGLS and RVFWS values predicted survival independent of other parameters at 6 months [hazard ratio (HR) = 0.37 (0.15–0.90), p = 0.02 and HR = 0.42 (0.19–0.89), p = 0.02; per 1 standard deviation increase respectively].

RVGLS proved to be a significant independent predictor of the 24 months mortality [HR = 0.53 (0.32–0.86), p = 0.01], and RVFWS showed a strong tendency [HR = 0.64 (0.40–1.00), p = 0.05]. RVFAC [HR = 0.67 (0.42–1.06), p = 0.09] failed to predict mortality at 24 months, TAPSE remained statistically significant [HR = 0.58 (0.35–0.96), p = 0.03].

In the second multivariable model (M2) we included the potential influential clinical covariates according to current ESC guidelines (NYHA class III/IV; QRS duration, LBBB; and medical treatment: BB, ACEi/ARB and AA intake). In this model, RVGLS predicted the 24 months mortality [HR = 0.55 (0.33–0.91), p = 0.01], but RVFWS did not [HR = 0.63 (0.40–1.00), p = 0.05], as shown in Table 5.

**Table 5:** Multivariable Cox regression analysis of right ventricular functional parameters predicting mortality at 6 and 24 months. Model 2 (M2): covariates were selected based on current CRT guidelines.

	M2								
	mortality at 6 months					mortality at 24 months			
	HR	95% CI	χ^2^	p		HR	95% CI	χ^2^	p
**RVGLS**	0.29	0.07–1.15	3.09	0.07	**RVGLS**	0.55	0.33–0.91	5.25	**0.02**
**RVFWS**	0.26	0.07–0.96	4.06	**0.04**	**RVFWS**	0.63	0.40–1.00	3.84	0.050
** **				** **	**RVFAC**	0.63	0.39–1.03	3.27	0.07
** **				** **	**TAPSE**	0.63	0.37–1.05	3.04	0.08

Hazard ratios refer to 1 standard deviation increase. Models were adjusted for the following covariates: NYHA II/III stadium, QRS duration, LBBB morphology, beta blocker, ACE inhibitor/ ARB and aldosterone antagonist intake. HR: hazard ratio, CI: confidence interval, χ^2^: wald chi square, OR: odds ratio, RVGLS: right ventricular global longitudinal strain.

**Fig 1:**
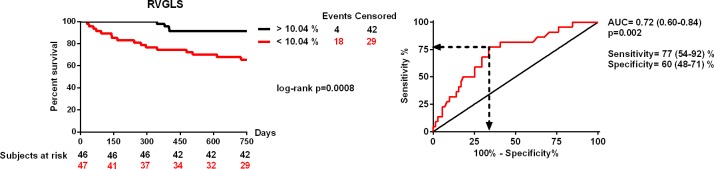
Receiver operating characteristics analysis and Kaplan-Meyer survival curves of patients with right ventricular global longitudinal strain below and above 10.04%.

The 24-months survival was significantly impaired in patients with RVGLS below 10.04% before CRT implantation [area under the curve = 0.72 (0.60–0.84), p = 0.002, sensitivity: 77 (54–92) %, specificity: 60 (48–71) %; log-rank p = 0.0008; HR = 5.23 (1.76–15.48), p = 0.003], as shown in Fig 1.

## Discussion

Synopsis of the key findings

Our study revealed that CRT improved right ventricular dysfunction by increasing RVGLS and RVFWS. Decreased baseline RVGLS was associated with poor short-term and long-term prognosis independent of other parameters, RVFWS showed only a tendency for mortality prediction. RVGLS below 10.04% before CRT implantation was associated with a 5-fold increased risk of 24-months mortality Conventional RV parameters, TAPSE and RVFAC affected prognosis moderately and changed in a statistically indifferent manner over time.

### Possible mechanisms and explanations

Right ventricular dysfunction has been associated with advanced heart failure. However, only few studies have investigated the effect of RV function in CRT implanted patients. The complex shape and volume dependency of the RV make it difficult to quantify RV morphology and function accurately using standard echocardiography. As a result, cardiac magnetic resonance (cMR) imaging has become the gold standard method to assess the RV [18].

The use of conventional 2D echocardiography (as a widely available and easy to obtain imaging method) remains a clinical challenge for the assessment of RV function, however TAPSE is still considered capable of quantifying RV longitudinal function [19]. A retrospective analysis of 848 CRT recipients demonstrated that after a median follow-up of 44 months, TAPSE ≤ 14mm was an independent predictor of all-cause mortality [7]. Similarly, a sub-study of the Cardiac Resynchronisation-Heart Failure (CARE-HF) trial investigated RV function in 688 patients, of whom 345 were assigned to CRT and lower TAPSE values were associated with higher mortality [20]. In our study, we were able to confirm these results. On the other hand, TAPSE represents only one aspect of the complex RV function and is strongly influenced by the overall heart motion and loading conditions as well as technical challenges. This could be the main reason why we measured statistically indifferent TAPSE values during the 6 months follow-up, despite the obvious changes of the RV strain parameters.

As a result of the inherent limitations of the TAPSE measurement, other indices of RV performance have been developed. For instance, in a small investigation of 77 CRT patients, the baseline RV myocardial performance index (RV MPI), (calculated as the ratio of isovolumetric contraction and relaxation times to ejection time) was associated with higher mortality and heart transplantation rate [21]. Similarly, Vizzardi et al. followed-up 93 CRT patients with moderate heart failure for five years and found that RV MPI predicts all-cause mortality and HF hospitalisation [19]. Another interesting parameter, the RV fractional area change (RVFAC) was also tested as a prognostic marker. Cameli et al. in 41 patients referred to heart transplantation found RVFAC to be an independent predictor of outcome [16]. Furthermore, in a retrospective analysis of the MADIT-CRT trial, every 5% increase in RVFAC was associated with 22% reduction in event rates and the lowest mortality rate was observed in patients who showed the best RV function at 1-year follow-up [22]. We also found lower RVFAC values in patients who died before 24 months, however RVFAC was not significantly influenced by CRT, suggesting the same problem that occurred with TAPSE measurement.

Nevertheless, a non-Doppler two-dimensional echocardiographic technology, the speckle tracking imaging has recently appeared, allowing for the quantification of global and regional deformations. As the RV myocardium is composed of mainly longitudinal fibres, (which are responsible for longitudinal shortenings occurring in the ejection period), the RV global longitudinal strain might be a promising non-invasive parameter to quantify RV function. In agreement with this, Cameli et al. investigated 41 patients before heart transplantation and RVGLS was found to be an independent predictor of outcome [16]. Furthermore, a recent study of Sade et al. followed up 120 patients after CRT implantation for a period of 5 years and determined RV function by using TAPSE, RVFAC, RVGLS and RVFWS. In accordance with our data, these authors found that RV dysfunction, defined as RVFWS < 18%, independently predicted long-term mortality with high specificity and sensitivity [8].

In our analysis we used an RVGLS cut-off value of 10.04%, which indicates that patients with slightly reduced RV function may also benefit from CRT implantation. Moreover, our study is the first to demonstrate that baseline RV function has prognostic relevance in predicting short-term mortality, and we found that patients with severely reduced RV function would not gain survival benefit from implanted CRT. Our study also suggests that RV speckle tracking imaging would be a better option to characterize RV dysfunction as compared with conventional methods.

It should therefore be important to precisely identify patients with RV dysfunction using the best modalities, since these patients not only have an elevated mortality risk, but are also more probably exposed to unnecessary surgical risks. On the other hand, a CRT implantation causes pointless increase in healthcare costs, when more effective therapies may allow better allocation of appropriate resources for the management of chronic heart failure.

## Limitations

Our study was a single centre, observational study with relatively low sample size and inherent limitations. First, since strain imaging highly depends on frame rate and image resolution, endocardial border tracing had to be performed manually. Second, as dedicated software for RV 2D strain was not available, thus we used a scheme designed for speckle tracking of the LV. Third, we obtained our data from apical 4 chamber view, as this was the only image plane for longitudinal RV deformation. Our echocardiographic protocol did not include 3D acquisitions, therefore, data on RV volumes and ejection fraction could not be acquired. Furthermore, RV diastolic function analysis was not performed. In addition, the RV strain might also be influenced by loading conditions, although in a lesser manner than by conventional RV measurements. Based on the above, our study was unable to include all relevant factors affecting the outcome of CRT, i.e. laboratory parameters such as NT-proBNP, which may have an effect on the results.

In view of these limitations, the presented results should be considered as being preliminary and hypothesis-generating, and future studies should be conducted in order to confirm our results. Despite these limitations, we believe our findings might be of remarkable clinical interest.

## Conclusion

Our analysis revealed that baseline RV dysfunction, characterized by strain parameters, is associated with poor prognosis after CRT implantation.

## Supporting Information

S1 Dataset Original data of patient characteristics and measurements. (ZIP)
